# Young adolescents' independent mobility, related factors and association with transport to school. A cross-sectional study

**DOI:** 10.1186/1471-2458-10-635

**Published:** 2010-10-22

**Authors:** Klara Johansson, Marie Hasselberg, Lucie Laflamme

**Affiliations:** 1 Department of Public Health Sciences, Division of Global Health/IHCAR, Karolinska Institutet, Nobels väg 9, SE-171 77 Stockholm, Sweden

## Abstract

**Background:**

Children's independent mobility differs between groups of adolescents, but knowledge is lacking on how mobility-limiting factors interact. This study explores the association between factors that can affect young adolescents' mobility, searching for typical patterns within a geographical area where mobility is both relatively high and promoted (in this case Stockholm County, Sweden). An additional question is how clusters of limiting factors and demographic attributes relate to active commuting to school.

**Methods:**

A sample of 7th grade students (ca 13-14 years old) in Stockholm County, Sweden, answered a survey (n = 1008). A cluster analysis was performed on variables descriptive of the respondents and of potential limitations to their independent mobility, such as fears, coping, traffic situation in the neighbourhood and parent/child opinions on mobility (18 variables and 50 categories). Active commuting to/from school was compared using proportion (with 95% confidence intervals) by cluster.

**Results:**

Five consistent and distinct clusters were identified. Among the most discriminating factors were fears experienced in the neighbourhood, strategies to cope with fear, type of housing and traffic environment. Girls were over-represented in the two clusters most typical of respondents experiencing fears (either several of these or darkness in particular) and boys in two others where housing (house vs. apartment) and neighbourhood conditions played a more determinant role. The proportion of active commuting among respondents was quite similar over clusters but was nonetheless higher in the cluster (over girls) reporting more fears and other factors limiting mobility.

**Conclusions:**

Whereas fears - and coping - are more typical of adolescent girls in the formation of the clusters, household and neighbourhood characteristics are more typical of boys. Broadly speaking, there seem to be two groups of girls with fears but these differ based on types of fear, ways of coping with fear and their living conditions. The association between the limitations to mobility and active commuting is unclear, the latter being higher among those disclosing a broader range of limiting factors, including fears.

## Background

Children's independent mobility increases with age [1,2], and in early adolescence they can be allowed to commute and move relatively freely, at least during daytime. Whereas there can be variations between and within countries, this description reflects well the situation in North European countries in general and in Stockholm in particular, where the current study was conducted [3,4]. Differences between adolescent groups in terms of what parents allow them to do (also called mobility *licenses *[5]*) *are not only regionally based but also vary by gender, with boys often being allowed to move more freely, and by other sociodemographic characteristics, including ethnicity [1,2].

Besides mobility licenses, adolescents' mobility may also be affected by their own fears and other perceptions of the environment [2], as well as by extrinsic factors like traffic intensity and other physical barriers in the environment [6]. These sources of influence also play a different role based on adolescents' sociodemographic characteristics, e.g. girls report more fears in the local environment [2,4].

Whereas different limiting factors are likely to be associated with one another, e.g. the character of the local environment and parents' attitudes towards their offspring's mobility [7], research in that domain is scarse. Likewise, whereas a number of sociodemographic attributes can be linked to more restricted mobility in some settings - e.g., female gender and ethnic minority [2] - it is unclear what the sociodemographic patterns of mobility restrictions are.

The main question addressed in this study is that of the association between factors that can affect young adolescents' mobility, and their sociodemographic characteristics, searching for typical patterns, within a geographical area where mobility is both relatively high and promoted (in this case Stockholm County, Sweden).

A second question addressed is how clusters of limiting factors and demographic attributes relate, in turn, to active commuting, a poorly researched question. Active commuting, for instance by walking or cycling to school, is known to vary across sociodemographic groups and environments [8]. Among younger children, it can be less common if, for instance, parents worry about the child's safety [9]. This does not apply to adolescents, where studies show no association between parents' perception of traffic safety in the neighbourhood and active commuting to school, for children aged 10-12 [10], 6-14 [11] or 5-18 years old [7]. More active commuting is associated with male sex [12], belonging to an ethnic minority [12], no car ownership in the family [11] and shorter distance to school [11].

## Methods

Data was gathered from a survey on mobility conducted among 7^th ^grade adolescents (i.e. aged 13 years ±1) in Stockholm County (Sweden) during the academic year 2005/06. Stockholm County includes all the municipalities in the greater Stockholm area, including the city, suburbs and some rural areas. From a random sample of 70 schools, a total of 44 agreed to participate (one regular class or one larger group of students per school) (see also [4]). Of 1,299 students invited, 1,009 agreed to participate (response rate of 77.6%; 79.7% among girls and 75.6% among boys). Questionnaires were self-administered on school computers during class time and group sessions were supervised by teachers or members of the research team.

Prior to the data collection, a letter was given to students with information regarding the study, including that participation was voluntary and that they could withdraw at any time. Written consent was requested from parents, and oral consent from students. The study was approved by the *Regional Ethical Review Board in Stockholm *(*Regionala etikprövningsnämnden i Stockholm, EPN*) dnr 2005/821-31.

The original purpose of the survey was to explore social differences in mobility, exposure to traffic risk and perceived safety and security, and therefore the questionnaire encompassed a broad range of questions related to mobility, commuting to school, fears and coping in the neighbourhood and the physical and social environment in the neighbourhood (see Additional file 1).

**Figure 1:**
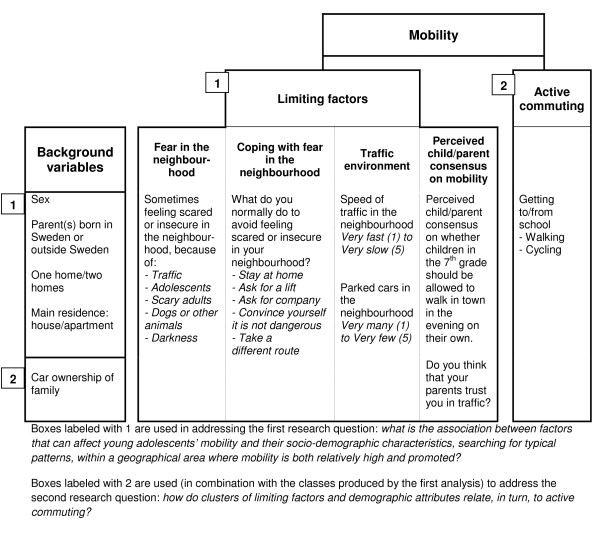
**Concepts and variables used in the analysis. All variables are self-reported**.

Figure 1 presents the data considered for this specific study.

Research question 1 - clusters of factors limiting individual mobility

Besides various sociodemographic attributes of the respondents (first column in Figure 1), attention was paid to feeling scared or insecure in one's own neighbourhood as well as to coping strategies. Indeed, adolescents' strategies to cope with fear may limit their mobility - such as choosing to stay at home or take another route - or to some extent facilitate mobility, such asking for company [4,13,14]. Fear was explored by asking "*Do you sometimes feel scared or unsafe in the area where you live as a result of one or more of the following things*?" Eight fixed options were available with one open response option. The eight options and the open responses were aggregated to the five fears in Table 1 (for more details on data treatment see Johansson et al [4]). Coping strategies were explored by asking *"What do you normally do to avoid feeling scared or insecure in your neighbourhood?" *with the response options in Table 1. Respondents were given the opportunity to respond to this even if they had not reported fear.

For fears and coping strategies, 'no response' was coded as 'no' to each specific fear or strategy (this was in accordance with how the question was phrased). For other variables in the analysis, 'no response' was coded together with "I don't know", where relevant.

An additional area of study was the traffic situation in the local environment. Respondents were asked to grade traffic speed in the living area on a five point scale, herein aggregated in three. The number of parked cars has been shown to be associated to adolescents' perception of their neighbourhood as unsafe or not a good place to grow up in [6]. Many parked cars may also constitute a physical barrier in the environment - and an injury risk factor for pedestrians and cyclists. Respondents graded number of parked cars on a five point scale.

A further aspect considered was the existence of consensus regarding mobility licenses between parents and children. "Perceived child/parent consensus on children's mobility" is a variable combining parallel questions on respondents' opinions on what children their age should be allowed to do on their own in terms of independent mobility and what they thought their parents' opinions were (see Table 1). Only opinions regarding "walking in town in the evening" were retained, to avoid overlap between variables and since this was the most controversial question. Another approach on parents' views on adolescent mobility was the question on whether respondents believed their parents trusted them in traffic.

During the selection process of variables for the study areas above, the variables too highly correlated were excluded (such as amount of traffic, which was closely related to speed of traffic; or additional questions on parent/child opinion on mobility in different situations in daytime/evening).

To highlight how the various limiting and sociodemographic factors considered related to one another, a cluster analysis was performed. The coded values of the variables in Table 1 (18 variables; 50 categories) were analyzed simultaneously, using a classification method called the Hierarchical Ascendant Classification (HAC) [15-17]. The HAC [15] is a cluster analysis method suitable for the treatment of categorical data as is the case herein. In the statistical software used (SPAD version 6.5), it is suggested that the HAC is applied following a Factorial Analysis of Correspondence (FAC) [16,17] performed on the original categorical data. The combined use of the methods in injury studies has been described in greater detail elsewhere [18] and later applications in safety studies can be found from road traffic settings [19,20].

The HAC divides the respondents into clusters so that every respondent belongs to one and only one cluster [17]. The respondents are classified based on their resemblance, i.e., their proximity, estimated by the chi-square metric. After the ascendant hierarchical system of clusters has been produced, four criteria are used to decide on what level of the hierarchy (how many clusters) to present the results. Low intra-cluster variance, or *compactedness *of a cluster, indicates high similarity of the individuals forming a cluster. The inter-cluster variance, or *separateness *of the clusters, indicates how distinct neighbouring clusters are from one another. The HAC aims to minimize the variance *within *clusters and maximize the variance *between *them. The third criterion is the *consistency *in the interpretation of clusters, which refers to the information contained in a cluster, in terms of the categories that have the most significant contribution to its formation (p < 0.05). If one - or several - cluster(s) is not very informative, one can consider moving up or down in the cluster hierarchy. The last criterion is the *informational benefit *of moving down or up in the hierarchy.

In this study, the HAC was performed on the first six factors of the FAC, i.e., using the coordinates of the variables analyzed on the first six factorial axes.

Research question 2: relationship with active commuting.

The second research question was dealt with by considering the clusters resulting from the analysis and the information on respondents' active commuting to and from school during the week (walking or cycling); and whether their family owned a car.

For each question (walking, cycling and car ownership), the proportion by cluster was presented with 95% confidence intervals.

## Results

Five consistent and distinct clusters of children were identified. Their main characteristics are highlighted in Table 1, where categories significantly over-represented in a cluster are marked in italics (i.e. using the chi-square metric mentioned above). The cluster descriptions that follow are based on these significant categories and the most typical ones are used to label the clusters.

**Table 1:** Description of the five clusters resulting from the Hierarchical Ascendant Classification (HAC)

Variables (^a^)	Categories	Cluster 1 (n = 361)	Cluster 2 (n = 223)	Cluster 3 (n = 186)	Cluster 4 (n = 127)	Cluster 5 (n = 111)	Total (n = 1008)
Socio-demographics		%	%	%	%	%	%

Gender (19.2%)	Female	32.4	40.8	***95.2***	***61.4***	45.0	50.9
	Male	***67.6***	***59.2***	4.8	38.6	55.0	49.1

Main residence (20.0%)	Apartment	8.0	***89.2***	26.3	***60.6***	***53.2***	41.0
	House	***91.1***	10.8	***72.6***	37.8	43.2	57.9
	Other/No answer	0.8	0.0	1.1	1.6	3.6	1.1

Parent(s) born outside Sweden (14.4%)	None	***79.5***	49.3	***77.4***	33.9	55.0	64.0
	At least one	20.5	***50.2***	22.6	***66.1***	44.1	35.8
	No answer	0.0	0.5	0.0	0.0	0.9	0.2

Living alternately with divorced parents (4.2%)	One home	***84.5***	63.2	76.9	80.3	74.8	76.8
	Two homes	15.5	***36.8***	23.1	19.7	25.2	23.2

Factors affecting mobility							

Parked cars in the area (49.6%)	1 (many)	1.4	***46.6***	7.5	***22.8***	10.8	16.3
	2	8.9	***17.5***	15.1	***20.5***	6.3	13.1
	3	17.5	18.4	18.8	24.4	10.8	18.1
	4	***37.4***	12.1	***30.7***	15.7	5.4	24.3
	5 (no parked cars)	***34.9***	5.4	23.7	7.9	8.1	19.9
	Don't know/no answer	0.0	0.0	4.3	8.7	***58.6***	8.3

Traffic speed in the living area (42.4%)	1 + 2 (rapid)	12.7	***23.8***	18.3	***33.1***	5.4	18.0
	3 (neither rapid nor slow)	25.2	***36.8***	***37.1***	29.9	10.8	29.0
	4 + 5 (slow traffic)	***62.1***	39.5	43.6	34.7	17.1	45.2
	Don't know/no answer	0.0	0.0	1.1	2.4	***66.7***	7.8

Sometimes feels scared or insecure in the neighbourhood, because of:							

Darkness (13.9%)	Yes	7.5	7.2	***54.8***	***50.4***	15.3	22.4
	No	***92.5***	***92.8***	45.2	49.6	84.7	77.6

Adolescents (13.7%)	Yes	6.9	11.7	23.1	***78.0***	10.8	20.3
	No	***93.1***	***88.3***	76.9	22.1	***89.2***	79.7

Adults (11.3%)	Yes	8.3	18.8	24.2	***63.0***	11.7	20.8
	No	***91.7***	81.2	75.8	37.0	***88.3***	79.2

Traffic (11.2%)	Yes	4.7	7.2	19.9	***56.7***	8.1	15.0
	No	***95.3***	***92.8***	80.1	43.3	***91.9***	85.0

Animals (10.7%)	Yes	3.1	7.2	2.7	***33.9***	9.9	8.5
	No	***97.0***	92.8	***97.3***	66.1	90.1	91.5

Strategies to cope with insecurity in the neighbourhood							

Ask for company (21.3%)	Yes	1.4	7.6	***69.4***	***46.5***	18.0	22.8
	No	***98.6***	***92.4***	30.7	53.5	82.0	77.2

Ask for a lift (15.3%)	Yes	2.5	0.9	***38.7***	***33.9***	9.0	13.5
	No	***97.5***	***99.1***	61.3	66.1	91.0	86.5

Convince oneself it is not dangerous (10.4%)	Yes	8.6	14.4	***52.2***	***49.6***	9.9	23.2
	No	***91.4***	***85.7***	47.9	50.4	***90.1***	76.8

Take a different route (8.9%)	Yes	4.2	2.2	***20.4***	***48.0***	6.3	12.5
	No	***95.8***	***97.8***	79.6	52.0	***93.7***	87.5

Stay at home (8.5%)	Yes	1.9	1.4	2.7	***39.4***	3.6	6.9
	No	***98.1***	***98.7***	***97.3***	60.6	96.4	93.2

Perceived child/parent^b ^consensus on walking in the city in the evening (15.2%)	Both yes	9.7	***14.8***	6.5	3.2	11.7	9.6
	Both no	22.2	16.6	***36.0***	***36.2***	18.9	24.9
	Child yes, parent no	7.2	***13.0***	7.0	8.7	5.4	8.4
	Child more conservative than parent	6.1	1.8	4.3	4.7	***12.6***	5.4
	Child yes, parent maybe/no answer	10.8	14.8	5.4	9.5	14.4	10.9
	Child maybe/no answer, parent no	23.0	17.0	25.3	13.4	16.2	20.1
	Both maybe/no answer	21.1	22.0	15.6	24.4	20.7	20.6

Do you think that your parents trust you in traffic? (9.7%)	Yes, always	***72.3***	***77.6***	***76.3***	40.2	55.0	68.3
	Yes, almost always	21.3	17.0	20.4	28.4	22.5	21.2
	Other	6.4	5.4	3.2	***31.5***	***22.5***	10.5

The categories of the variables that significantly contributed to the formation of each cluster are marked in italics.

Note: numbers are rounded and may add upp to more than 100%.

^a ^Cumulated contribution to the first 3 factors within parenthesis.

^b ^Parents' opinions reported by child

### Cluster 1

*Living in a house and not requesting company as a means to feel safe (35.8% of the respondents; 15.2% intra-cluster variance)*. Typical of this first group is that over 90% live in a house and almost all report not asking for company to cope with fear in their neighbourhood. There is an overrepresentation of respondents not reporting fear of darkness, and also of those not reporting fear of other adolescents, adults, traffic or animals. For coping strategies, in addition to not reporting asking for company, there is an overrepresentation of not using the strategies of convincing themselves there is no danger nor asking for a ride, as well as not taking a different route nor staying at home. Two respondents in three are boys. One third say that there are no parked cars in their neighbourhood and in addition, more than a third say that there are only few parked cars. Two thirds say that traffic in the area is slow. There is an overrepresentation of respondents with both parents born in Sweden, who only live in one place, and who believe their parents always trust them in traffic.

### Cluster 2

*Living in an apartment, in an area with many parked cars (22.1% of the respondents; 11.2% intra-cluster variance)*. Most characteristic of the respondents in this cluster is living in an apartment and reporting many (or quite many) parked cars in their neighbourhood. There is an overrepresentation of respondents who choose not to use the strategies of asking for a ride, asking for company, taking another route and staying at home, or trying to convince themselves there is nothing to be afraid of. For fears, what is typical for this cluster is to not report fear of darkness, and also to not report fear of traffic or of adolescents.

Respondents who report two homes (living alternately with divorced parents) and those with at least one parent born outside Sweden are overrepresented. 78% think their parents always trust them in traffic. 59% are boys. Almost 37% report traffic in their area to be neither rapid nor slow and an additional 24% report it to be rapid. There is an overrepresentation of respondents who report that both they and their parents think that adolescents in 7^th ^grade should be allowed to walk in town after dark and also of those who report that they think so but that their parents don't.

### Cluster 3

*Asking for company to feel safe, being a girl, reporting fear of darkness, asking for a ride and trying to convince themselves there is no danger (18.5% of the respondents; 10% intra-cluster variance)*. In this group, what is most characteristic is that almost 70% of respondents report that they ask for company to feel safe in their neighbourhood, over 95% are girls, more than half report fear of darkness; and asking for a ride or trying to convince themselves there is no danger is also much overrepresented. Another coping strategy that is overrepresented is taking a different route - but there is also an overrepresentation of not using the strategy of staying at home. There is an overrepresentation of respondents who live in a house and whose parents were both born in Sweden. More than expected by chance report that both they and their parents think adolescents in 7^th ^grade should not be allowed to walk in town in the evening.

Respondents reporting no fear of dogs or other animals in their neighbourhood are overrepresented. More than expected by chance say that their parents always trust them in traffic, that traffic in their area is neither rapid nor slow and that there are few cars parked in the area.

### Cluster 4

*Fear of adolescents, adults and traffic and coping by staying at home or changing route (12.6% of the respondents; 10.9% intra-cluster variance)*. What characterizes this group is that more than expected report fear of other adolescents in their area, use the strategy of staying at home, report fear of traffic, fear of adults and that they use the strategy of taking a different route. Respondents reporting fear of animals and fear of darkness, and coping with fear by trying to convince themselves there is no danger, asking for a ride or asking for company are also overrepresented. More than expected by chance report at least one parent born outside of Sweden. Those who do not think that their parents always or almost always trust them in traffic are overrepresented. More than expected by chance live in an apartment, think that traffic in the area is fast, and say they and their parents agree that 7^th^-graders should not be allowed to walk in town after dark. 61.4% of the respondents in this group are girls. More than expected by chance report that there are many or quite many cars parked in their area.

### Cluster 5

*No opinion on either traffic speed or number of parked cars (11.0% of the respondents; 7.5% intra-cluster variance)*. More than anything else, this group is characterised by the fact that the majority don't know whether traffic is rapid or slow or whether there are many parked cars. Respondents who did not report that their parents trust them in traffic are overrepresented. Those who report not using the strategy of convincing themselves there is no danger, as well as not taking a different route are also overrepresented. There is an overrepresentation of those who are inclined to be more conservative than they consider their parents would be in their attitude towards allowing children their age to walk in the city after dark, and also of not reporting fear of adolescents, adults or traffic. More than expected by chance live in an apartment.

### Summary of clusters

To sum up, from the descriptions presented above, it appears that in clusters where girls are over-represented, mobility is more a reflection of how the respondents feel when moving in their neighbourhood whereas in clusters where boys are over-represented, housing conditions and the traffic environment play a primary role. Of course, as has been seen, several other parameters come into play to differentiate the clusters from one another. Yet, some labels could be proposed to summarize and distinguish them: Cluster 1, "suburban house residents and independent"; Cluster 2, "residents of apartment in areas with high traffic density"; Cluster 3 "girls afraid of darkness"; Cluster 4, "fearful with hindered independent mobility"; and Cluster 5, "no opinion on the traffic environment".

### Active commuting

Whereas 60.6% of all respondents walked to and/or from school only 14.2% cycled. As seen in Table 2, only children from Cluster 4 walked in a significantly higher proportion, with 75.4% walking. Although adolescents in Cluster 1 cycled more than those from the other clusters, the difference was not significant.

**Table 2:** Cluster-specific proportion of respondents actively commuting to school (with 95% confidence intervals)

	Cluster 1	Cluster 2	Cluster 3	Cluster 4	Cluster 5	Total
	n = 361	n = 223	n = 186	n = 127	n = 111	n = 1008
	% (95% CI)	% (95% CI)	% (95% CI)	% (95% CI)	% (95% CI)	% (95% CI)
Transport to and/or from school:						
Walk	58.1 (53.0 - 63.2)	57.0 (50.4 - 63.5)	57.0 (49.8 - 64.1)	75.4 (67.8 - 83.0)	64.9 (55.9 - 73.8)	60.6 (57.5 - 63.6)
Cycle	19.6 (15.4 - 23.7)	10.8 (6.7 - 14.8)	10.2 (5.8 - 14.6)	10.3 (5.0 - 15.7)	15.3 (8.6 - 22.1)	14.2 (12.1 - 16.4)

No car in the family	5.0 (2.7 - 7.2)	22.9 (17.3 - 28.4)	5.4 (2.1 - 8.6)	22.0 (14.8 - 29.3)	15.3 (8.6 - 22.1)	12.3 (10.3 - 14.3)

By contrast, not having a car in the family (12.3% in total) varies a lot across clusters, with higher - and similar - frequencies in Clusters 2 and 4 and lower in Clusters 1 and 3 (Table 2).

## Discussion

Five clusters were highlighted, out of which four were more typical of either girls or boys (two clusters each). For girls, fears and strategies to cope with fears were determining factors. In cluster 4 (labelled "fearful with hindered independent mobility"), fears were many and diverse (adolescents, adults and traffic), and coping strategies implied limitations to mobility (staying at home or modifying one's route). Together with those characteristics, we also found an over-representation of respondents with parent(s) born outside Sweden or living in an apartment. In prior studies these are all factors that have been associated with fear and restricted mobility [1,2,4,21,22]. Also with the other cluster of girls (Cluster 3, labelled girls afraid of darkness) this does not come as a surprise as girls usually express more fear of darkness [4,23]. What distinguishes these two clusters is primarily how they cope with these fears, their housing conditions and whether their parents are Swedish born or not.

In the case of boys, living in a house (Cluster 1) or in an apartment (Cluster 2) is very discriminating. In Sweden, a majority of those living in a house own their home, implying good socioeconomic conditions [21], and in Stockholm, it generally implies living outside the city centre. As opposed to the clusters more typical of girls, those clusters display far fewer fears and greater mobility. As indicated in earlier studies, male sex and, to some extent, living in a house and having Swedish-born parent(s) are related to reports of fewer fears and enhanced mobility [1,2,4,21], which applies well to the respondents from Cluster 1 (labeled "suburban house residents and independent"). Although from a different environment, respondents from Cluster 2 (labeled "residents of apartment in areas with high traffic density"), were also below average on most fears and all coping strategies. Their living areas seem to be of higher urban density - apartment, more parked cars and high traffic speed. In Stockholm, areas of this kind can be found both in the city centre and in different types of suburbs, and these areas can reflect a wide range in social status.

For their part, parents' opinions on mobility and the consensus between child and parent came out as a less significant determinant in the formation of the clusters. Interestingly however, for the cluster formed of girls afraid of darkness (Cluster 3) as well as the fearful with hindered independent mobility (Cluster 4), the category where both child and parent are *negative *to adolescent mobility in town in the evening was over-represented while the category where both child and parent or children alone are *positive *was over-represented in Cluster 2 (residents of apartment in areas with high traffic density). Characteristic of Cluster 4 ("fearful with hindered independent mobility") was also the opinion that parents don't trust the adolescents in traffic. This can be an indication of differences in parenting style, which in turn can be a reflection of the character of their local (traffic) environment.

From among the five clusters identified, one was rather unclear (Cluster 5), since their most determining factors were not reporting either traffic speed or cars parked in their environment. It is possible that these respondents were not very interested in the survey but they would not differ much from other students in that respect. To a question at the end of the questionnaire (see additional file 1) about half of them (54%) answered that they thought it was less than "fun or interesting" to take the survey, which was not significantly different from the average for the other clusters (42%).

Regarding active commuting, walking was by far the most common way to get to school in all groups. Proportions were significantly more common among the "fearful with hindered independent mobility" (Cluster 4). One possible explanation is that some respondents from that group may live in an area with high urban density, as indicated by their reports on their area and household conditions, which in turn can be linked with shorter distance to school. Alternatively, as access to car is less common in this group, opportunities to get a lift to school may be fewer. Another interpretation is that the "fearful with hindered independent mobility" respondents may have formed a group that reported fears and other mobility limitations precisely because walking to school made them and their parents aware of the risks and barriers in their environment.

### Strengths and limitations

The data collection strategy and materials at hand have three major advantages. Even though several schools dropped out, the study sample included a large number of schools (44) and these were spread out across different types of environments within Stockholm County. Likewise, the number of students participating in the survey was substantial (over 1,000) and came from a variety of schools and living areas. Further, the information gathered was based on self-reports, which is preferable to proxy-reports by parents, in particular for the age group concerned. Though we originally selected a random sample of schools, data collection took place only in those first 44 schools (from an original sample of 70) that accepted to take part in the survey. For the question under study herein, there is no obvious indication that this convenience sample of schools and the classes in each school chosen for participation would differ in any determinant ways from those not participating. It is therefore reasonable to believe that the results presented herein are accurate at school level.

Respondents were clustered within schools, which could result in some groups of adolescents being oversampled and produce Type I errors if observations within clusters are correlated [24]. However, the number of schools is relatively high, which decreases the risk of clustering.

Fear- and coping related questions were explorative. They covered several potential sources but none of them in depth. Many questions were inspired by a literature review and some pre-tests but our coverage might still be limited in scope. Open answers suggest for instance that coping strategies may include talking on a mobile phone or listening to music as a strategy to avoid fear.

There might be some desirability bias due to gender stereotypes, i.e. boys might be less willing to disclose for instance strategies like "asking for company" or "asking for a ride", even though they might feel more secure in the company of friends or adults.

Parents' licences and restrictions have come out as less prominent in this study. Whereas it might be related to the respondents' age group, it is possible that our use and treatment of the information masked some details of interest. We used those aspects where there was the greatest parent-child disparity.

## Conclusions

Whereas fears and coping strategies are more typical of adolescent girls in the formation of the clusters, household and traffic characteristics in the neighbourhood are more typical of boys. In the two clusters where fears occur, the coping strategies typically used indicate different levels of impeded independent mobility. There are no major indications that, in the clusters more representative of boys, housing and traffic conditions would be linked to some experience of altered mobility. The association between those clusters and active commuting is not self-evident, the latter being higher among those disclosing a broader range of limiting factors, above all various fears.

## Competing interests

The authors declare that they have no competing interests.

## Authors' contributions

KJ coordinated and carried out development of the survey design and the questionnaire, carried out the data collection, participated in data analysis and drafted the manuscript. MH participated in development of the survey design and the questionnaire, helped with the data collection and helped draft the manuscript. LL participated in development of the survey design and the questionnaire, carried out data analysis and helped draft the manuscript. All authors have read and approved the final manuscript.

## Pre-publication history

The pre-publication history for this paper can be accessed here:

http://www.biomedcentral.com/1471-2458/10/635/prepub

## Supplementary Material

Additional file 1 **English translation of survey questionnaire "Adolescents' encounter with traffic"**. An English translation of the questionnaire in Swedish which was used in the survey "Adolescents' encounter with traffic", among 7^th ^grade students in a sample of schools in Stockholm County, 2005/06. Click here for file
