# Supplementary material for: Young adolescents' independent mobility, related factors and association with transport to school. A cross-sectional study
Source: BMC Public Health. 2010 Oct 22;10:635. doi: 10.1186/1471-2458-10-635 (PMC2978150; doi:10.1186/1471-2458-10-635)
Supplement: Additional file 1 — English translation of survey questionnaire "Adolescents' encounter with traffic". An English translation of the questionnaire in Swedish which was used in the survey "Adolescents' encounter with traffic", among 7th grade students in a sample of schools in Stockholm County, 2005/06. [file 1471-2458-10-635-S1.DOC]

### Survey questionnaire: Adolescents’ encounter with traffic – English translation

This is an English translation of the survey questionnaire “Ungdomars möte med trafiken” (Adolescents’ encounter with traffic) that was done among 7^th^ grade students in Stockholm County, 2005/06.

The survey was in Swedish and has not been used in English.

The survey was web-based and students responded on school computers, during class time.

Please note that this is only the text of the questionnaire, and does not reflect the lay-out, which was designed to facilitate understanding.

Thank you for answering this questionnaire,

Please do not write your name - your answers are to be anonymous.

No one at school will see your questionnaire once it’s completed.

Read the questions in order and try to answer as honestly as possible.

Q1 In which year were you born?

1. 1990
2. 1991
3. 1992
4. 1993
5. 1994

Q2 Are you...

1. Girl
2. Boy

Q3 In which country were you born?

1. Sweden
2. Other. Which?

Q4 In which country were your parents born?

Fill this in about the persons you think of as your real parents. If you only have one parent, just fill in for that one.

1. Parent 1
2. Parent 2

Q5 Do you live in one or two places?

(Sometimes children and teenagers live in more than one place, for example if they’ve got divorced parents.)

1. I live in one place
2. I live in two places

(Based on which alternative respondents choose, those who live in one place were automatically sent to answer Q6-Q12 and those who live in two places, to Q13-Q21 and Q33).

Here are a few questions about how you live.

Q6 What is your post code?

Example: 120 47 Enskede gård

(If you’ve forgotten one or several numbers write X instead)

Q7 What kind of house do you live in?

1. An apartment
2. A row-house
3. A detached house
4. Other, what: ____________

Q8 Do you have your own room?

1. No
2. Yes

Q9 Does your family have a computer at home?

1. No
2. Yes. How many?

Q10 Does your family own a car?

Pick-ups, minivans and other similar vehicles also count.

1. No
2. Yes, one vehicle.
3. Yes, two or more.

Q11 How long have you lived in this area?

1. I moved here this year.
2. I have lived here for 1-5 years.
3. I have lived here for more than 5 years.

Q12 How many adults and children live at your house, in your family? (do NOT include yourself)

1. Number of parents or other adults________ (18 years old or more)
2. Number of older siblings or other children that are the same age as you, or older_______ that usually live here.
3. Number of younger siblings or children younger than you______ that usually live here.

**You answered that you live in two places. We will first ask you how it is where you live this week.**

Q13 What is your postcode where you live at the moment?

Q14 How often do you live here?

1. Most of the time
2. Half the time
3. Regularly, but less than half of the time
4. Rarely

Q15 What kind of house is the house you’re living in this week?

1. An apartment
2. A row-house
3. A detached house
4. Other. What?

Q16 Do you have your own room?

1. No
2. Yes

Q17 Does your family have a computer at home?

1. No
2. Yes. How many?

Q18 Does your family own a car?

Pick-ups, minivans and other similar vehicles also count.

1. No
2. Yes, one vehicle.
3. Yes, two or more.

Q19 How long have you lived in this area?

1. I moved here this year
2. I have lived here for 1-5 years
3. I have lived here for more than 5 years

Q20 How many adults and children live at your house, in your family? (do NOT include yourself)

1. Number of parents or other adults________ (18 years old or more)
2. Number of older siblings or other children that are the same age as you, or older_______ that usually live here.
3. Number of younger siblings or children younger than you______ that usually live here.

**We will now ask you about your second home.**

Q21 What postcode does your other home have?

**Now, more questions about the home where you live now.**

On a scale from 1-5, how would you describe the traffic in your neighbourhood?

Think about what it looks like outside your house and on the streets all around and mark the number you think suits your neighbourhood the best.

Q22 The traffic:

| Heavy traffic |  |  |  | Light traffic | I don’t know |
| --- | --- | --- | --- | --- | --- |
| 1 | 2 | 3 | 4 | 5 |  |

Q23 The traffic:

| Very fast |  |  |  | Very slow | I don’t know |
| --- | --- | --- | --- | --- | --- |
| 1 | 2 | 3 | 4 | 5 |  |

Q24 The car drivers:

| No cars stop at zebra crossings when I want to cross |  |  |  | All cars stop at zebra crossings when I want to cross | I don’t know |
| --- | --- | --- | --- | --- | --- |
| 1 | 2 | 3 | 4 | 5 |  |

Q25 Parked cars:

| A lot of cars are parked on the streets |  |  |  | No cars are parked on the streets | I don’t know |
| --- | --- | --- | --- | --- | --- |
| 1 | 2 | 3 | 4 | 5 |  |

Q26 Do you think your parents trust you when you’re by yourself in traffic?

| No, never |  |  |  | Yes, always | I don’t know |
| --- | --- | --- | --- | --- | --- |
| 1 | 2 | 3 | 4 | 5 |  |

If you want to add something, you may write this in the box below.

Q27 How do you like your neighbourhood?

| Not at all |  |  |  | Very much | I don’t know |
| --- | --- | --- | --- | --- | --- |
| 1 | 2 | 3 | 4 | 5 |  |

Q28 Do you sometimes feel scared or unsafe in the area where you live as a result of one or more of the following things?

(Here you can choose more than one alternative and if none of them feels right, don’t choose any)

1. Car traffic
2. Mopeds and motorcycles
3. Cyclists
4. Same-age adolescents
5. Older adolescents
6. Scary adults
7. Dogs or other animals
8. Darkness
9. I don’t know
10. Something else, feel free to tell us what:

Q29 What do you usually do to not feel scared or unsafe where you live?

(You can choose more than one alternative)

1. I don’t usually feel so scared or unsafe.
2. I usually stay at home
3. I usually ask someone to drive me
4. I usually ask someone to come with me or meet me on the way
5. I usually tell myself that there’s nothing to be scared of
6. I usually take a different route
7. I can’t remember
8. I don’t often come up with anything to do
9. There is nothing to do
10. Something else. What?

Q30 Have you or your friends ever been subjected to any of the following things in your neighbourhood?

1. To be hit
2. Someone had taken something from you
3. To be chased
4. If you want to tell us something about this, you can write in the box:

Q31 How would you describe your neighbourhood with three words?

Q32 Tell us something about...

1. Fun things there are to do in your neighbourhood or:
2. Fun things you would like to be able to do in your neighbourhood.

Q33 Is there anything that is different in the other area where you live?

(Tell us if you want)

Q34 How are you mostly getting to school and going home from school this week?

(Choose one answer for ”to school” and one for ”home from school”)

1. I’m walking to school
2. I’m walking home from school
3. I’m cycling to school
4. I’m cycling home from school
5. I’m going by car to school
6. I’m going by car from school
7. I’m going by bus, train, commuter train, subway or tram to school
8. I’m going by bus, train, commuter train, subway or tram home from school
9. Some other or several other ways to school:
10. Some other or several other ways to school:

Q35 How long does it take you to get to school?

1. Less than 5 minutes
2. 5-15 minutes
3. 15-30 minutes
4. 30 minutes-an hour
5. Longer than an hour
6. I don’t know

Q36 If you could choose, how would you prefer to get to school?

1. My usual way
2. In another way:

Q37 How would you describe your journey to school with three words?

Q38 Do any of the options below bother you on your way to school?

(Again you can choose more than one option, and if none of them bother you don’t need to choose any)

1. Car traffic
2. Mopeds and motor-cycles
3. Cyclists
4. Same-age adolescents
5. Older adolescents
6. Scary adults
7. Dogs or other animals
8. Darkness
9. I don’t know
10. Something else, feel free tell us what:

Q39 What do you enjoy doing most in your free time?

Q40 Think about yesterday. Did you do any of the things below, and if you did - how long did it take?

|  | Yes, less than 20 minutes | Yes, more than 20 minutes | No | I don’t know |
| --- | --- | --- | --- | --- |
| Travel by car  Cycle in traffic  Walk in traffic  Use in-lines, roller skates, skateboard or a scooter in traffic  Travel by bus, subway, commuter train or tram | 🞎  🞎  🞎  🞎  🞎 | 🞎  🞎  🞎  🞎  🞎 | 🞎  🞎  🞎  🞎  🞎 | 🞎  🞎  🞎  🞎  🞎 |

Q41 If you think about last Saturday, did you do anything of the things below? If you did, how long time did it take?

|  | Yes, less than 20 minutes | Yes, more than 20 minutes | No | I don’t know |
| --- | --- | --- | --- | --- |
| Travel by car  Cycle in traffic  Walk in traffic  Use in-lines, roller skates, skateboard or a scooter in traffic  Travel by bus, subway, commuter train or tram | 🞎  🞎  🞎  🞎  🞎 | 🞎  🞎  🞎  🞎  🞎 | 🞎  🞎  🞎  🞎  🞎 | 🞎  🞎  🞎  🞎  🞎 |

Q42 Are there any things you would like to do in your free time but that you can’t do or you’re not allowed to do?

1. Yes
2. No
3. I don’t know

[Question 43 was only asked to those who responded “Yes” to Q42.]

Q43 You have said there’s something you can’t or are not allowed to do. Why?

Q44 Do you have a bike?

1. Yes
2. No
3. I share a bike with someone else

Q45 Do you have a bus/subway pass for use on Stockholm public transport?

1. Yes
2. I share it with my family
3. No

Q46 Approximately how much money of your own do you have every month, which you can decide how to spend?

Q47 What should the money cover?

You may fill in more than one alternative.

1. Clothes
2. Trips in Stockholm
3. Toiletries (such as make-up and hair products)
4. Books and/or computer games
5. Cinema, concerts, football games etc.
6. Candy, coffees etc.
7. Other. Give examples.

Q48 Do you think you have more or less money than your schoolmates?

1. I have more money
2. I have the same amount as most others
3. I have less money
4. I don’t know

Q49 Have you ever been in a traffic accident?

(As a pedestrian, cyclist, in a car or another vehicle.)

1. Yes
2. No
3. I can’t remember

If yes, you can share something about that, if you want to:

Q50 Have you ever witnessed a traffic accident?

1. Yes (if you want, tell us about it)
2. No
3. I can’t remember

If yes, you can share something about that, if you want to:

Q51 How hard/easy is it to make your way around your neighbourhood?

1. When you walk

| Very hard |  |  |  | Very easy | I don’t know |
| --- | --- | --- | --- | --- | --- |
| 1 | 2 | 3 | 4 | 5 |  |

1. When you cycle

| Very hard |  |  |  | Very easy | I don’t know |
| --- | --- | --- | --- | --- | --- |
| 1 | 2 | 3 | 4 | 5 |  |

Q52 How do you think it is to travel to other neighbourhoods?

1. When walking

| Very hard |  |  |  | Very easy | I don’t know |
| --- | --- | --- | --- | --- | --- |
| 1 | 2 | 3 | 4 | 5 |  |

1. When cycling

| Very hard |  |  |  | Very easy | I don’t know |
| --- | --- | --- | --- | --- | --- |
| 1 | 2 | 3 | 4 | 5 |  |

1. When you travel by bus, subway, commuter train or tram

| Very hard |  |  |  | Very easy | I don’t know |
| --- | --- | --- | --- | --- | --- |
| 1 | 2 | 3 | 4 | 5 |  |

Q53 Are there any of the options below that make your travelling more difficult?

You may fill in more than one alternative.

|  | In daytime | In the evening |
| --- | --- | --- |
| Cars | 🞎 | 🞎 |
| Mopeds, motorcycles | 🞎 | 🞎 |
| Cyclists | 🞎 | 🞎 |
| Same-age adolescents | 🞎 | 🞎 |
| Older adolescents | 🞎 | 🞎 |
| Unpleasant adults | 🞎 | 🞎 |
| Dogs or other animals | 🞎 | 🞎 |
| Lack of street lamps | 🞎 | 🞎 |
| Lack of buses, trains etc | 🞎 | 🞎 |
| Too expensive | 🞎 | 🞎 |
| Something else, give an example if you want | 🞎 | 🞎 |

Q54 Tell us if there’s something you do to make it easier when you have a hard time getting around.

Q55 What do you think is appropriate for a 7th grader to do on their own?

Fill in one or more examples.

|  | Yes | No | Maybe |
| --- | --- | --- | --- |
| 1. Use the subway in daytime 2. Use the subway in the evening 3. Travel by bus in daytime 4. Travel by bus in the evening 5. Cycle in daytime 6. Cycle in the evening 7. Walk around in town in daytime 8. Walk around in town in the evening | 🞎  🞎  🞎  🞎  🞎  🞎  🞎  🞎 | 🞎  🞎  🞎  🞎  🞎  🞎  🞎  🞎 | 🞎  🞎  🞎  🞎  🞎  🞎  🞎  🞎 |

Q56 What do your parents think is appropriate to do on your own as a 7th grader?

|  | Yes | No | Maybe |
| --- | --- | --- | --- |
| 1. Use the subway in daytime 2. Use the subway in the evening 3. Travel by bus in daytime 4. Travel by bus in the evening 5. Cycle in daytime 6. Cycle in the evening 7. Walk around in town in daytime 8. Walk around in town in the evening | 🞎  🞎  🞎  🞎  🞎  🞎  🞎  🞎 | 🞎  🞎  🞎  🞎  🞎  🞎  🞎  🞎 | 🞎  🞎  🞎  🞎  🞎  🞎  🞎  🞎 |

LAST PAGE

Q57 If there is anything else you want to tell us about your neighbourhood, please share it with us.

Q58 What do you think about answering this questionnaire?

1. Very fun or interesting
2. Quite fun or interesting
3. Not much
4. Quite boring or hard
5. Very boring or hard

Q59 On a scale from 1-5, how easy or difficult was it to answer...

1. ...the questions about the traffic yesterday?
2. ...the questions about the traffic in your neighbourhood?
3. ...the questions about whether your parents trust you in traffic?
4. ...the questions about whether there are things you would like to do but can’t?
5. ...the questions about whether you’re bothered about anything on the way to shool?

If you have a comment, write it in the box:

1. Bad

2.

3.

4.

1. Good

Q60 We’d like you to write a comment about the whole questionnaire.

**Thanks for your help!**

You’ve now completed the questionnaire. If you’re satisfied with your answers, press ”done”.

Your answers have been saved, thanks for your participation.
